# Plasma transthyretin is a nutritional biomarker in human morbidities

**DOI:** 10.1007/s11684-022-0940-3

**Published:** 2022-08-09

**Authors:** Yves Ingenbleek

**Affiliations:** Faculty of Pharmacy, Laboratory of Nutrition, University of Strasbourg, Route du Rhin, Illkirch-Graffenstaden, F-67401 Strasbourg, France

**Keywords:** lean body mass, nutritional status, transthyretin, malnutrition, inflammation, amyloidosis

## Abstract

Transthyretin (TTR) is a small liver-secreted plasma protein that shows close correlations with changes in lean body mass (LBM) during the entire human lifespan and agglomerates the bulk of nitrogen (N)-containing substrates, hence constituting the cornerstone of body building. Amino acids (AAs) dietary restriction causes inhibition of TTR production and impairs the accretion of LBM reserves. Inflammatory disorders result in cytokine-induced abrogation of TTR synthesis and urinary leakage of nitrogenous catabolites. Taken together, the data indicate that malnutrition and inflammation may similarly suppress the production of TTR through distinct and unrelated pathophysiological mechanisms while operating in concert to downsize LBM stores. The hepatic synthesis of TTR integrates both machineries, acting as a marker of reduced LBM resources still available for defense and repair processes. TTR operates as a universal surrogate analyte that allows for the grading of residual LBM capacity to reflect disease burden. Measurement of TTR is a simple, rapid, and inexpensive micro-method that may be reproduced on a daily basis, hence ideally suited for the follow-up of the most intricated clinical situations and as a reliable predictor of any morbidity outcome.

## Introduction

Protein and calorie malnutrition (PCM) is associated with the phylogeny of mankind. During centuries, observation of declining anthropometric parameters, mainly growth retardation and body weight (BW) loss, was regarded as indicative of a poor health state. Quetelet was the first to propose in 1835 a scoring formula associating 2 biometric measures to grade in adult subjects the level of a ponderal deficit or an excessive overweight marred by increased mortality risk [1]. In children, there are many clinical outcomes of dietary deficiencies whose extreme poles are kwashiorkor (KW) and marasmus (MR). The first clinical observation of KW was described in 1933 by Cecily Williams who was working as a pediatrician in Accra, the capital of the Republic of Ghana (formerly Gold Coast). She reported that the condition occurred some weeks after the weaning period when the flourishing breast-fed toddler was abruptly transferred to a protein-depleted plant regimen [2]. The Ghanaian KW denomination was adopted by the World Health Organization (WHO) in 1952 [3]. Analytical electrophoresis of blood from children with PCM revealed reduced concentrations of serum albumin (Alb) and of many other circulating proteins [4]. In addition to the classical anthropometric measures used at that time to grade malnutrition, Alb became the gold standard biochemical indicator of PCM in the following two decades. KW is mainly the result of misguided weaning counseling that affects not only underprivileged population groups but also well-educated parents living in affluent countries, notably those influenced to implement extravagant vegan regimens [5]. MR is a disease typically affecting neonates born from mothers dying during the delivery process when neither a surrogate mother nor bottle-feeding is available. Nutritional disorders mainly result from long-term deprivation of protein and energy intake counterbalanced by increased mobilization of fat and muscle reserves leading to severe stunting and height retardation [6]. Children with MR usually survive for months owing to efficient adaptive mechanisms, but the lifespan may be shortened by the coexistence of drought, famine, civil wars, and population displacements [6]. MR may also affect teenagers, adults, and elderly persons. A high prevalence of several forms of PCM was identified at the end of the seventies in hospital settings, both in medical [7] and surgical [8] wards. Here, current anthropometry and measurement of Alb were the criteria used for the follow-up of these hospital patients.

## TTR as nutritional biomarker

The discovery of TTR, also named prealbumin, as nutritional biomarker has an unexpected background. At the beginning of the 1970s, the Directory Board of the renowned Institute of Cellular & Molecular Pathology (ICMP, Catholic University of Louvain, Belgium) led by Professor Christian De Duve, winner of Nobel Prize in Physiology or Medicine, made the decision to conduct collaborative studies with African countries. After having received full agreement from the Pediatrics Department of the University of Dakar (Senegal, West Africa), the project was entrusted to Prof. M. De Visscher, head of the ICMP Endocrine Unit. The objective was to launch a comprehensive investigation covering all aspects of thyroid function in malnourished children, including intestinal malabsorption and kinetics of iodine in bodily tissues; thyroid uptake, compartmental distribution and clearance; and the synthesis, secretion and plasma transport of thyroid hormones. The exploration started with the analysis of the three carrier-proteins of thyroid hormones, namely thyro-binding globulin (TBG) [9], Alb, and TTR. The analysis of TTR was performed using a radial immunodiffusion procedure with partigen immunoplates (Behringwerke, A.G., Germany) containing anti-TTR antibodies. The study revealed the following conclusions: (1) Both TTR and Alb indices changed in the same direction during the active stage of PCM as well as during the recovery period but with steeper slopes for TTR, indicating its higher level of sensitivity to protein depletion and repletion. (2) For unknown reasons at that time, TTR plasma concentrations were found to be systemically more depressed in children with KW than in those with MR. The main data from this first study were published in 1972 [10], stressing the particular importance of TTR for the early detection of marginal PCM states and for the follow-up and outcome of any severe nutritional disorder. These investigations, once completed, became the topic of a Ph.D. dissertation that was successfully defended at the ICMP in 1977 [11].

## Body composition studies

There are several methodological approaches for the determination of body composition. In healthy young men (20–25 years old) with a BW of 70 kg, the fat mass (FM) represents 18% of BW, whereas LBM amounts to 82% of BW and comprises the fat free mass (FFM) which contains 20% of body proteins and mineral mass (MM) which includes the bulk of elemental abundance (11% of BW) [12]. With increasing age, physiologic alterations occur in the body composition of healthy elderly persons (60–70 years old) with a 10% gain in FM (18% to 28%) and a 10% loss in LBM (82% to 72%) [12]. Pioneering studies performed by Forbes using dual-energy X-ray absorptiometry (DXA) for the measurement of $^{40}K$ has rapidly emerged as the method of choice for assessing the size of LBM regardless of age and disease state [12]. DXA is grounded in the fact that at least 95% of the naturally occurring nonradioactive potassium ($^{39}K$) remains confined within the intracellular space of all bodily organs together with minute amounts (0.0117%) of $^{40}K$, the major source of natural β-radioactivity in living tissues. $^{39}K$ and $^{40}K$ isotopes have interchangeable turnover activities, maintaining close interrelationships with nitrogenous (N) compounds [13]. Fig. 1 outlines the evolutionary patterns of total body K (TBK) throughout the human lifespan allowing for the calculation of the LBM in both sexes from birth until very old age [12]. LBM comprises a composite agglomeration of fat-free organs and tissues that may schematically be subdivided into a visceral compartment that includes organs characterized by rapid metabolic turnover rates (liver, intestinal mucosa, thymoleukocytic tissues, brain) and a structural compartment that is distinguished by slower turnover rates (skeletal musculature (SM), skin, connective and cartilaginous appendages). The liver, the chief organ of the visceral compartment accounts for approximately 2.6% of BW (1.8 kg in healthy adult men) and has an oxygen consumption rate of 44 mL $O_2$/kg [14]. SM, the main tissue of the structural compartment accounts for 38% of BW (approximately 30 kg in healthy adult men) [15] and has an oxygen consumption rate of 2.3 mL $O_2$/kg [14]. The data show that the 20-fold faster protein turnover rate in the liver is counterpoised by the nearly 20-fold heavier SM weight, indicating that these organs equally contribute to one quarter (26.4% vs. 24.6%) of the basal heat production of the body, usually defined as resting energy expenditure (REE). More recent

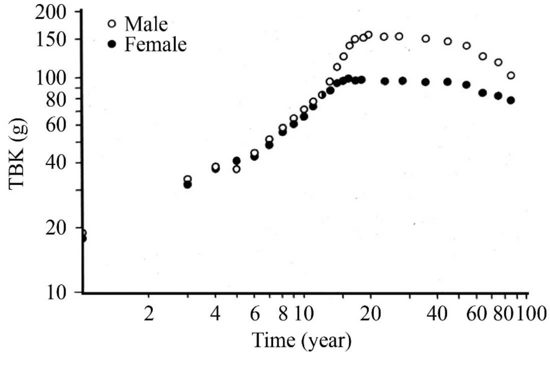

**Fig. 1** Body accretion of TBK values during the lifespan of healthy human subjects. TBK, total body K.

investigations have validated these interrelationships recorded 75 years ago, revealing nevertheless lower hepatic and SM values of 19.9% and 18.4%, respectively [16]. In PCM patients, particular attention must be paid to the intestinal mucosa (10% of total REE) [17] and to thymoleukocytic tissues (7% of total REE) [18]. The brain deserves special consideration as it is a small organ weighing ~1.4 kg that is made up primarily of fat (60%) rather than N-containing compounds (20%); the latter portions is the site of very active oxidation processes fed by continuing blood glucose fluxes resulting in cerebral heat production. Clinical investigations have highlighted that, despite this imbalanced disparity, the brain is not metabolically dependent upon FM but related to FFM [19]. The REE value generated by cerebral tissues has been calculated as 24.3% [19] in agreement with previous studies [20], making this organ a heat supplier at least equivalent to the liver or SM. Overall, it appears that the sum of the total production of heat by these LBM organs and tissues accounts for approximately three quarters of the total body REE cost. This rate of heat production may be markedly altered at any time when organ(s) and tissue(s) are affected by protein-depleted states.

The whole body assessment of TBK levels was achieved using DXA technology which allows the measurement in biological tissues of the naturally occurring β-radioactivity of $^{40}K$. The pioneering devices were assembled at the University of Rochester, New York, USA, under the expert guidance of G.B. Forbes [12] in close collaboration with the International Atomic Energy Agency (IAEA), Vienna, Austria. Fig. 1 compiles 7 different clinical investigations performed in healthy subjects from birth until very old age. The results are plotted against age in double-logarithmic coordinates. The bulk of TBK (95%) sequestered within metabolically active tissues is tightly correlated with total body N (TBN), making this last parameter a reliable tool to assess LBM values in health and disease. The data show a linear progression without sexual differences from birth until the onset of puberty, abrupt S-shaped rising trajectory artifically dampened during adolescence owing to altered graduations of the abscissa scale, occurrence of gender dimorphism with plateau levels during adulthood, and disappearance of sexual differences after the sixth decade [12].

## TTR in healthy subjects and protein-depleted states

TTR is a highly conserved protein that is secreted by the liver in the bloodstream of birds and eutherian mammals [21]. The human TTR gene has been localized in the long arm of chromosome 18q [22]. TTR has a homotetrameric unglycosylated structure whose molecular mass reaches 55 kDa. The four subunits comprise 127 AAs each [23] with one monomer conveying a small companion protein displaying a single binding site for one molecule of all-*trans*-retinol, hence its retinol binding protein (RBP, 21 kDa) denomination [24]. Binding of holo-RBP to TTR occurs in the liver before its extracellular exportation in the form of a retinol circulating complex (RCC, 76 kDa) [25]. Despite their different biological half-lives (2 days for TTR [26] vs. half a day for RBP [27]), both TTR and RBP molecules remain attached at close 1:1:1 stoichiometry [25]. TTR safeguards RBP from premature urinary output and serves as a limiting factor for the delivery of retinoid compounds to target tissues [28].

TTR was identified in human cerebrospinal fluid in 1942 [29] and in human serum in 1956 [30]. The protein is detected in the fetal blood 8 weeks after conception [31] and is operative during the early steps of embryogenesis. At birth, the plasma TTR level is approximately two thirds of that measured in healthy mothers and increases thereafter linearly without sexual differences during infant growth [32]. Human puberty is characterized by major hormonal and metabolic alterations promoting the development of SM in male teenagers whereas estrogens result in less enlargement of the female musculature [33]. As a result, a hormonally-induced sex dimorphism occurs at the onset of adolescence with significantly higher S-shaped elevation of TTR recorded in male adolescents compared with the blunted curve documented in teenaged girls [34] (Fig. 2). In healthy adults, the sex-related difference in plasma TTR values is maintained in the form of plateaued levels during full sexual maturity [34]. Normal TTR concentrations are stabilized at approximately 300–330 mg/L in adult males and at approximately 250–270 mg/L in adult females. Senescence is characterized by physiologic involution of SM in both sexes starting from the sixth decade but with a steeper slope in elderly males, explaining that TTR plasma values no longer exhibit sexual difference once individuals reach their eighties [34].

TTR concentrations were measured in the blood samples of 68 720 healthy US citizens from birth until very old age using immunoturbidimetric analysis [34]. Plasma TTR values identify the accretion and/or losses of N in bodily tissues, conferring to the biomarker the unique property to reflect the fluctuations of LBM stores

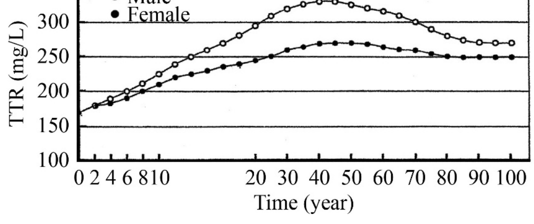

**Fig. 2** Evolution of plasma TTR concentrations throughout healthy human lifespan. TTR, transthyretin.

in health and disease. The more pronounced elevation of TTR values in adolescent males is in keeping with the androgen-induced development of a larger skeletal musculature. Sexual dimorphism and TTR plateau levels observed during adulthood disappear after the sixties, indicating that elderly men and women undergo physiologic sarcopenia stages with no longer sexual differences. Decline in plasma TTR values remains correlative with the ongoing non-physiologic sarcopenia. Both TTR and sarcopenia biomarkers work as predictors of mortality risk owing to the reduced LBM stores impairing the nutritional and inflammatory responses associated with stressful disorders.

Normal growth of neonates from birth until adulthood implies continuing accretion of body proteins and is synonymous with positive N balance leading to LBM expansion. These growth processes are tightly regulated and require the intake of appropriate energy and AA building blocks [35]. Plasma TTR is obviously situated on the cutting edge of the energy/AA balance, and their optimal requirements depend upon age, physiologic status and disease conditions which need compensation for increased deficiency-induced losses [36]. Restriction of dietary AA supply leads to curtailed N balance and to unachieved LBM replenishment. This is accompanied by depressed hepatic production of TTR mRNA [37], decreased abundance of TTR nuclear transcripts [38] and corresponding reduced exportation of mature TTR molecules into the bloodstream. The early decline in TTR plasma values is attributed to the exquisitely sensitive liver response to protein-depleted states, to the small TTR pool size and to its short biological half-life ($T_{1/2.}$) [39]. As expected, TTR has been proposed as an indicator of dietary energy/protein adequacy in preterm, normal, and sick neonates [40,41], as a detector of marginal PCM in children [42], and as a predictor of outcome in adult [43] and geriatric [44] patients. In the absence of superimposed inflammatory stresses, a drop in plasma TTR reflects an unbalanced provision of nutrient classes impairing the accretion of N in LBM compartments and setting adaptive mechanisms in motion which largely precede the development of biochemical or clinical symptoms of malnutrition.

KW disease is characterized by desquamating skin; heavy liver steatosis replacing up to 50% of the normal parenchymal tissue [45] with downgraded Alb production resulting in anasarca; regressive flattening of the intestinal mucosa [46] causing severe malabsorptive syndromes and watery diarrhea; high vulnerability to infectious complications [47] imposing high mortality rates; and alterations in consciousness and behavior together with anorexia likely resulting from defective activities of dopamine and serotonin neurotransmitters [48]. In contrast, MR patients reveal severe SM shrinkage, exhibiting signs of muscle wasting. They have less pronounced fatty liver, no edema [6], and relatively well preserved intestinal, immune, and cerebral functions. Taken together, the data show that LBM stores undergo distinct changes during the course of PCM morbidity progression. Whereas KW patients demonstrate concomitant protein depletion in the four principal LBM compartments resulting in marked decrease in TTR plasma levels, MR patients are enduring grievous sarcopenia but minimal damage in the three other LBM compartments explaining moderately depressed TTR values. Normal plasma concentrations measured in healthy subjects indicate that the LBM stores have attained full replenishment using homeostatic mechanisms [39]. In PCM patients, the decreased TTR values represent a lack of N-containing molecules required to restore full LBM reserves. The actual TTR plasma level found in children with PCM appears to result from the sum of N-substrates lost in each LBM component, which explains the lower values found in children with KW than in those with MR. It becomes apparent that each LBM component, taken separately, may undergo N-depletion or recovery processes that can influence the hepatic level of TTR production. A good example of the close relationships linking LBM and TTR is provided in celiac children by the progressive involution of the intestinal mucosa correlated with declining TTR plasma values. By turn, administration of a gluten-free diet may be the trigger for mucosal recovery and TTR restoration [49].

Surveys taking into account sex- and age-differences have shown that the TTR marker exhibits a Gaussian distribution [11], paving the way for epidemiological approaches comparing health status between population groups. It is also worth noting that TTR is not affected by ethnic differences or genetics, as reported for other plasma proteins, notably for TBG in Australian natives [50]. The data provide additional support for the universal reliability of TTR as a nutritional biomarker.

## TTR and inflammatory disorders

Inflammatory disorders of any cause are initiated by activated leukocytes releasing cytokines that function as autocrine, paracrine, and endocrine molecules [51]. Proinflammatory cytokines stimulate the overproduction of counterregulatory hormones (glucocorticoids, catecholamines, glucagon, and growth hormone) and oppose the hypoglycemic and anabolic effects of insulin over-secretion, thus resulting in insulin resistance in healthy tissues [52]. Despite the presence of ambient hyperglycemia, maintenance of low respiratory quotient values (RQ ~0.7) indicates that the overall energy economy is based on the mobilization of fat stores, allowing the sparing of glucose and AA residues which are preferentially driven toward injured regions to carry out defense functions [52]. During the course of most chronic and acute inflammatory processes, several cytokines reorganize overall protein metabolism controlling the release of acute-phase reactants (APRs) which contribute in several ways to repairing injuries using specific kinetic and functional properties [53]. The severity and duration of the initial impact leads to increased protein breakdown which predominates over protein synthesis [54], indicating that the N balance is negative. The urinary analysis of N catabolites reveals that both visceral and structural compartments participate in the overall depletion of LBM stores in the whole body [52]. Interleukin-6 (IL-6) works as a key mediator that results in dramatic elevation of APR values [53] together with reciprocal suppression of TTR synthesis as demonstrated in animal [55] and clinical [56] experiments assuming molecular mechanisms completely distinct from those involved in protein-depleted states [37,38]. A position paper [57] raised doubt about the fundamental processes whereby nutritional and inflammatory factors work independently of each other to cause reduced hepatic syntheses of TTR. These opinions are grounded on the undocumented assumptions that any cytokine-induced disorder increases capillary permeability and generates liver reprioritization of protein synthesis that is redirected toward the preferential production of APRs at the expense of visceral proteins. This belief thus sustains the view that non-nutritional factors may impair the validity of TTR, hence depriving the marker of any nutritional relevance. The review [57] sponsored by the International Federation of Clinical Chemistry and Laboratory Medicine (IFCC), has resulted in a larger audience with endorsement of most Western societies of clinical nutrition, including the European and American Societies of Parenteral and Enteral Nutrition (ESPEN [58], ASPEN [59]). The high citation rate of the IFCC paper [57] has resulted in the use of TTR in clinical settings being largely discredited. It is an important time to consider molecular studies showing that the synthesis of TTR is defective in protein-depleted states [37,38] while undergoing blockade of TTR production in cytokine-induced tissue breakdown [55,56]. In other words, the reduced plasma level of TTR reflects the sum of dual distinct processes, yielding a marker grading the size of LBM stores still available for metabolic, immune and repair purposes as a net result. The data are consistent with the finding that TTR is the sole plasma protein adhering closely to fluctuations in LBM in health and disease [60,61]. The data imply that centrally-mediated regulatory mechanisms govern coordinated signaling systems maintaining the balance between protein depletion and protein accretion as well as interorgan fluxes between LBM components.

The assessment of the nutritional status of hospitalized Western patients started following the proposal made by Blackburn *et al.* [62] to measure Alb, a biomarker currently associated with the Quetelet formula (body mass index (BMI) is weight/squared height) together with mid-arm circumference (MAC), triceps skinfold thickness (TSF), and hand-grip strength (HGS). In the subsequent decades, clinical teams created approximately 20 novel scoring formulas with the aim of more accurately identifying the bodily changes occurring during the deterioration or healing of morbidities. A recently published review article enumerates with some details the parameters included in these formulas comprising classical immune, lipidic, and hematological data [63]. Table 1 provides a non-exhaustive listing of the most utilized formulas applied to critically ill patients and classified in alphabetical order without taking into account scientific precedence. The main criticism of these formulas is their lack of universality. Some of them may be adapted and helpful in some disease states but not in others. A good example of this limitation is described in a survey aiming at grading malnutrition in elderly patients using 6 different screening tools (GNRI, MNA, MUST, NRI, NRS, SGA) yielding dissonant results [64]. Another major weakness of these formulas lies in the fact that none of them is metabolically connected with the core of LBM, as it is the case for TTR [60,61], hence missing the cornerstone of body building and skimming only the surface of PCM-pathophysiology.

## Future prospects

In recent decades, the interest of clinicians in the above

**Table 1** Some scoring formulas used to assess the nutritional status of hospital patients

| |
|---|
| CONUT: prognostic COntrolling NUTritional status |
| FNA: Full Nutritional Assessment |
| GNRI: Geriatric Nutrition Risk Index |
| MI: Maastricht Index |
| MNA: Mini Nutritional Assessment |
| MST: Malnutrition Screening Tool |
| MUST: Malnutrition Universal Screening Tool |
| NRI: Nutritional Risk Index |
| NRS: Nutritional Risk Screening |
| NUTRIC: NUTrition RIsk in the Critically ill |
| PNI: Prognostic Nutritional Index |
| PNRS: Pediatric Nutritional Risk Score |
| PNST: Pediatric Nutrition Score Tool |
| SGA: Subjective Global Assessment |
| SNAQ: Short Nutrition Assessment Questionnaire |

formulas has progressively decreased mainly due to their poor clinical relevance and their variability depending on age, sex, and disease stages. The adoption of valid and reliable biomarkers of nutritional status is of importance for hospitalized patients, explaining a great deal of effort undertaken by Western societies of clinical nutrition to improve their current practice.

The goal was to develop new screening tools with global consensus among clinicians to define better categorization using criteria with worldwide impact and more adapted therapeutic strategies. The Global Leadership Initiative on Malnutrition (GLIM) was created in January 2016 appointing a core leadership committee and a GLIM working group gathering representatives of most international societies of clinical nutrition [65]. Priority was given to the diagnosis of malnutrition in hospitalized patients following a two-step procedure: (1) early identification of patients at risk of entering the malnutrition cycle and (2) assessment and grading of any declared malnutrition state. Five criteria were proposed to attain this basic objective: three phenotypic criteria (BW loss, low BMI, sarcopenia) and two etiologic criteria (reduced food intake, inflammatory burden). The most disturbing criterion is the inclusion of BMI which is subjected to two lines of criticism. The first is related to the inaccuracy of height measurements in all age groups [66] and especially in elderly persons or in patients who are in a wheelchair or bedridden [67]. The second refers, more importantly, to studies reminding that BW in the BMI formula results from the combination of FM and FFM, each item endowed with opposing influences marked by increased and decreased mortality risks, respectively [68,69]. In the BMI formula, high FM accretion rates may attenuate the protective role played by FFM values and thereby underestimate the risk of malnutrition while overestimating the lethality risk among overweight subjects [68,69]. These pathophysiological observations have incited Heymsfield himself, together with his Brazilian coworkers, to advise clinicians against using the BMI scoring formula in clinical settings [70].

The above data strongly support the view that the serial measurement of TTR allows monitoring of fluctuations in LBM depletion [60,61] and predicting the outcome of critically ill patients [71]. It is worth recalling that the TTR micro-method is simple, rapid, inexpensive, and reproducible. In the most complex clinical situations where malnutrition and inflammation are inextricably interwoven, such as burns, polytrauma, septicemic or neoplastic invasion, the serial measurement of TTR is ideally suited to identify the global health status of these patients on a daily basis. The follow-up of adult persons afflicted by less severe disease conditions may be achieved by TTR screening performed 2 or 3 times a week. The lower limit of the normal TTR range is approximately 200 mg/L, which is consistent with clinical investigations showing that below this cut-off level, the likelihood of serious complications may be increased [72,73]. In adult subjects, the threshold of 100 mg/L has ominous prognostic significance [74,75] likely due to exhaustion of LBM resources [60]. Cut-off values ranging from 200 mg/L to 100 mg/L thus define a gray zone between which TTR concentrations may fluctuate and predict either the best or the worst outcome. A recent prospective study using a receiver operating characteristic (ROC) curve of TTR values associated with malnutrition pointed to 170 mg/L and 120 mg/L for upper and lower cut-off lines, respectively [76]. These data reveal close links with the results recorded in clinical practice [72–75] despite the fact that the ROC investigation covers slightly reduced breadth values [76]. In malnourished children, the lowest TTR value compatible with survival has been estimated at 65 mg/L [77].

Elderly persons constitute a growing proportion of individuals with morbidities in our society, which deserves special consideration in health care programs. Fig. 3 indicates that the plateau levels measured for TTR throughout adulthood undergo progressive downregulation with stepwise disappearance of sexual differences after individuals reach their sixties. A recent publication provides details on this physiological sarcopenia associated with TTR decline during the last four decades of life, reaching 241 mg/L in healthy centenarian subjects [78]. This level of TTR is well above the turning point defining a possible morbidity risk, conferring to these elderly persons a good health safety margin. Obtaining such results requires regular consumption of a well-balanced diet, continuing physical activity, refreshing sleep and brain stimulatory activities. When the level decreases below the cut-off line

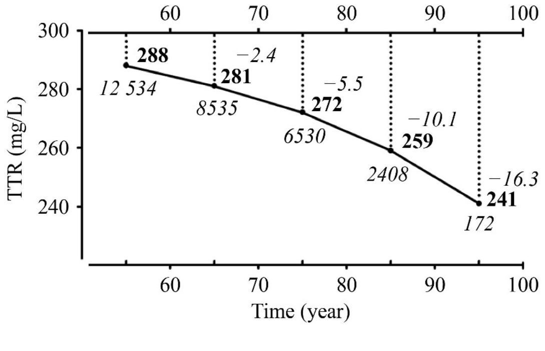

**Fig. 3** Evolutionary patterns of the physiologic sarcopenia in healthy centenarians. TTR values are measured in 17 645 healthy US elderly subjects aged 65 to 100 years (5796 men and 11 849 women) stratified into 4 decennial categories. Owing to the lack of sexual difference after the age of 65 years, mean TTR concentrations are combined and expressed in bold characters. Values indicated in italics represent the number of participants recorded in each decennial class. TTR measured in both sexes during the last four decades of life are situated well above the lower limit of normalcy, implying that these healthy centenarians do not incur life-threatening risks. TTR, transthyretin.

of 200 mg/L, TTR may serve as a warning signal indicating that something is going wrong, urging elderly persons to undergo deeper investigation even in the absence of complaints, clinical symptoms or questionable laboratory data. The situation may be the consequence of disrupted dietary balance, intestinal malabsorption, or low-grade inflammation. In elderly PCM patients, TTR values have the unique property to maintain high positive correlations ($r$ = 0.64) with LBM size [61] implying that any further plasma TTR decline is associated with additional losses of N-containing- substrates from their LBM tissue storage sites. The resulting non-physiologic sarcopenia is recognized as a major predictor of mortality in aged people [79]. In a large cohort of 7815 hemodialysis patients, the relative risk (RR) of lethality was calculated to be inversely related to serum TTR concentration [80]. The data show that both sarcopenia and TTR are equally informative parameters forecasting fatal outcomes as a result of depleted LBM reserves [78].

Developing adapted nutritional strategies may allow for two lines of response. Gradual TTR elevation rising above nadir levels indicates the reversal of N-balance and the progressive restoration of LBM resources. It may be the result of transient alleviation of the morbid process or the foretaste of the best possible clinical recovery. It is worth noting that increasing TTR values may occur when most other biological analytes still remain stable, pinpointing the unrivalled performance of TTR. In contrast, the maintenance of a steady TTR level reveals inappropriate therapeutic monitoring and/or harmful effects generated by underlying co-morbidities requiring specific managemental approaches. Using TTR as a routine screening assay allows early detection and better nutritional follow-up of high-risk patients while shortening the length of hospital stay and alleviating the financial burden of hospitalization [81].

## Amyloidosis

TTR may undergo substitutions of a single residue at different positions along the monomeric sequence. Replacement process of one AA by another may generate a wild-type of senile systemic amyloidosis affecting mainly elderly persons. Identification of the first molecular defect (Val30Met) causing a familial amyloidotic polyneuropathy (TTR-FAP) has autosomal dominant transmission and worldwide prevalence. Further investigations on these inherited TTR-FAP defects have led to the discovery of 3 geographically restricted endemic clusters (Portugal, Sweden, Japan) and of more than 150 other variants characterized by planetary distribution, variable penetrance, and clinical expression. The amyloidotic disorder is characterized by extracellular deposits of misfolded and insoluble fibrils and appearance of small globular oligomeric species in body tissues [82]. Besides sensorimotor disturbances stemming from cerebral and neural damages found in TTR-FAP patients, inherited variants may damage cardiac, gastro-intestinal, renal, vascular, and ocular tissues. Most amyloidotic patients manifest unintended weight loss, early satiety, diarrhea, hypoalbuminemia, and proteinuria. In the absence of treatment, the survival time ranges from 2 to 15 years. Clinical trials aiming at improving the disease outcome comprises orthotopic liver transplantation [83] and the use of therapeutic drugs such as Tafamidis [84] and Diflunisal [85] helping to stabilize and to prevent the misfolding of TTR molecules. More recently, researchers are experiencing novel classes of pharmacological agents with promising properties, notably Patisiran having the potential to target the 3′ untranslated region of TTR mRNA and to entail TTR knockdown [86] or Inotersen inhibiting TTR production through an antisense oligonucleotide approach [87]. A very large comparative review on clinical usefulness of Diflunisal, Tafamidis, Patisiran and Inotersen came to the conclusion that all 4 drugs were able, despite moderate adverse effects, to attenuate the severity of peripheral neuropathy, improve the quality of life, delay disease progression with survival benefit without halting the inexorable evolution toward fatal outcome [88].

The administration of Patisiran poses special nutritional and metabolic problems. The drop of mutant TTR to −80% of its starting value is accompanied by similar downregulation of the non-mutant TTR value and the 2 other components (RBP and retinol) of RCC, consistent with previous studies showing that the fall of plasma TTR works as a limiting factor for RBP transport and delivery of retinoid compounds to target tissues [28]. Owing to the fact that the duration of Patirisan treatment may reach 2 years, the consequences of a protracted thyroid and retinoid-deprived environment in TTR-AFP patients deserve to be maintained under surveillance, taking into account the major effects exerted by these hormonal ligands on the regulation of (glyco)protein synthesis and immune activities. The nutritional status of most amyloidotic patients undergoing above-mentioned pharmacological treatments is currently followed up using the modified BMI formula (mBMI = BMI × Alb) despite the fact that BMI may be problematic in clinical settings [68–70]. Moreover, Alb is characterized by a long biological half-life ($T_{1/2}$ ~20 days) explaining its poor sensitivity to detect alterations occurring in protein-depleted states. In the absence of TTR bioavailability, alternative approaches of nutritional status could be carried out by the measurement of LBM using surrogate DXA and UV absorbance technologies [89] or bioelectrical impedance analysis-derived phase angle [90].

## Concluding remarks

The use of TTR as a nutritional biomarker was first proposed half a century ago with the publication of the princeps paper in 1972 [10]. Clinical investigations and comparative surveys have raised countless debates and controversies in medical circles and academic institutions during the last decades. The peak of dissensus was reached in 2007 with the publication of a position paper [57] readily adopted by the Directory Boards of ASPEN and ESPEN, not mentioning the participation of many national societies of clinical nutrition affiliated with them. In recent years, a deluge of review articles was promulgated to promote the banishment of the TTR biomarker from nutritional studies [58,59,65].

In the meantime, the use of TTR is encouraged in most Asian countries such as China [72–74,91–94], Japan [75,95–99], and South Korea [100–102] focusing more specific attention on matters having major public health impact. Many other Eastern countries, notably Indonesia [103], Thailand [104], India [105], Iran [106], and Australia [107] are working along similar lines, having provided substantial contributions. The above data obviously indicate that the choice of the TTR biomarker recommended by many Eastern countries does not meet the GLIM guidelines prevailing in Western societies.

## Compliance with ethics guidelines

Yves Ingenbleek declares no conflict of interest. This manuscript is a review article and does not involve a research protocol requiring approval by the relevant institutional review board or ethics committee.

**Open Access** This article is licensed under a Creative Commons Attribution 4.0 International License, which permits use, sharing, adaptation, distribution and reproduction in any medium or format, as long as you give appropriate credit to the original author(s) and the source, provide a link to the Creative Commons license, and indicate if changes were made.

The images or other third party material in this article are included in the article's Creative Commons license, unless indicated otherwise in a credit line to the material. If material is not included in the article's Creative Commons license and your intended use is not permitted by statutory regulation or exceeds the permitted use, you will need to obtain permission directly from the copyright holder.

To view a copy of this license, visit https://creativecommons.org/licenses/by/4.0/.

## References

[1] Quetelet A (1835). Issue on Man and Development of his Faculties.

[2] Williams CD (1933). A nutritional disease of childhood associated with a maize diet. Arch Dis Child.

[3] Brock JF, Autret M (1952). Kwashiorkor in Africa. Bull World Health Organ.

[4] Anderson CG, Altmann A (1951). The electrophoretic serum-protein pattern in malignant malnutrition. Lancet.

[5] Chase HP, Kumar V, Caldwell RT, O’Brien D (1980). Kwashiorkor in the United States. Pediatrics.

[6] Akombi BJ, Agho KE, Hall JJ, Wali N, Renzaho AMN, Merom D (2017). Stunting, wasting and underweight in sub-saharian Africa: a systematic review. Int J Environ Res Public.

[7] Bistrian BR, Blackburn GL, Vitale J, Cochran D, Naylor J (1976). Prevalence of malnutrition in general medical patients. JAMA.

[8] Hill GL, Blackett RL, Pickford I, Burkinshaw L, Young GA, Warren JV, Schorah CJ, Morgan DB (1977). Malnutrition in surgical patients. An unrecognised problem. Lancet.

[9] Ingenbleek Y, De Nayer P, De Visscher M (1974). Thyroxine-binding globulin in infant protein-calorie malnutrition. J Clin Endocrinol Metab.

[10] Ingenbleek Y, De Visscher M, De Nayer P (1972). Measurement of prealbumin as index of protein-calorie malnutrition. Lancet.

[11] Ingenbleek Y (1977). Protein-calorie malnutrition in the child of lower age. Repercussions on thyroid function and serum carrier proteins.

[12] Forbes GB (1987). Human Body Composition.

[13] Cohn SH, Vartsky D, Yasumura S, Vaswani AN, Ellis KJ (1983). Indexes of body cell mass: nitrogen versus potassium. Am J Physiol.

[14] Brožek J, Grande F (1955). Body composition and basal metabolism in man: correlation analysis versus physiological approach. Hum Biol.

[15] Janssen I, Heymsfield SB, Wang ZM, Ross R (2000). Skeletal muscle mass and distribution in 468 men and women aged 18–88 yr. J Appl Physiol.

[16] Heymsfield SB, Müller MJ, Bosy-Westphal A, Thomas D, Shen W (2012). Human brain mass: similar body composition associations as observed across mammals. Am J Hum Biol.

[17] Nakshabendi IM, McKee R, Downie S, Russell RI, Rennie MJ (1999). Rates of small intestinal mucosal protein synthesis in human jejunum and ileum. Am J Physiol.

[18] McNurlan MA, Sandgren A, Hunter K, Essén P, Garlick PJ, Wernerman J (1996). Protein synthesis rates of skeletal muscle, lymphocytes, and albumin with stress hormone infusion in healthy man. Metabolism.

[19] Heymsfield SB, Peterson CM, Bourgeois B, Thomas DM, Gallagher D, Strauss B, Müller MJ, Bosy-Westphal A (2018). Human energy expenditure: advances in organ-tissue prediction models. Obes Rev.

[20] Elia M, Kinney JM, Tucker HN (1992). Organ and tissue contribution to metabolic rate. Energy Metabolism: Tissue Determinants and Cellular Corollaries.

[21] Power DM, Elias NP, Richardson SJ, Mendes J, Soares CM, Santos CR (2000). Evolution of the thyroid hormone-binding protein, transthyretin. Gen Comp Endocrinol.

[22] Wallace MR, Naylor SL, Kluve-Beckerman B, Long GL, McDonald L, Shows TB, Benson MD (1985). Localization of the human prealbumin gene to chromosome 18. Biochem Biophys Res Commun.

[23] Kanda Y, Goodman DS, Canfield RE, Morgan FJ (1974). The amino acid sequence of human plasma prealbumin. J Biol Chem.

[24] Kanai M, Raz A, Goodman DS (1968). Retinol-binding protein: the transport protein for vitamin A in human plasma. J Clin Invest.

[25] Monaco HL, Richardson SJ, Cody V (2009). The transthyretin-retinol binding protein complex. Recent Advances in Transthyretin Evolution, Structure and Biological Functions.

[26] Socolow EL, Woeber KA, Purdy RH, Holloway MT, Ingbar SH (1965). Preparation of I-131-labeled human serum prealbumin and its metabolism in normal and sick patients. J Clin Invest.

[27] Peterson PA, Nilsson SF, Ostberg L, Rask L, Vahlquist A (1975). Aspects of the metabolism of retinol-binding protein and retinol. Vitam Horm.

[28] Ingenbleek Y, Van Den Schrieck HG, De Nayer P, De Visscher M (1975). The role of retinol-binding protein in protein-calorie malnutrition. Metabolism.

[29] Kabat EA, Moore DH, Landow H (1942). An electrophoretic study of the protein components in cerebrospinal fluid and their relationship to serum proteins. J Clin Invest.

[30] Schönenberger M, Schultze HE, Schwick G (1956). A prealbumin of human serum. Biochem Z.

[31] Andreoli M, Robbins J (1962). Serum proteins and thyroxineprotein interaction in early human fetuses. J Clin Invest.

[32] Vahlquist A, Rask L, Peterson PA, Berg T (1975). The concentrations of retinol-binding protein, prealbumin, and transferrin in the sera of newly delivered mothers and children of various ages. Scand J Clin Lab Invest.

[33] Veldhuis JDL, Roemmich JN, Richmond EJ, Rogol AD, Lovejoy JC, Sheffield-Moore M, Mauras N, Bowers CY (2005). Endocrine control of body composition in infancy, childhood, and puberty. Endocr Rev.

[34] Bienvenu J, Jeppson JO, Ingenbleek Y. Transthyretin & retinol-binding protein. In: Ritchie RF, Navolotskaia O. Serum Proteins in Clinical Medicine. Foundation for Blood Research, Scarborough, Maine, 1996: 9.011–9.018

[35] Young VR, Yu YM, Fugakawa NK, Kinney JM, Tucker HN (1992). Energy and protein turnover. Energy, Metabolism, Tissue Determinants and Cellular Corollaries.

[36] Pencharz PB (2010). Protein and energy requirements for “optimal” catch-up growth. Eur J Clin Nutr.

[37] de Jong FA, Schreiber G (1987). Messenger RNA levels of plasma proteins in rat liver during protein depletion and refeeding. J Nutr.

[38] Straus DS, Marten NW, Hayden JM, Burke EJ (1994). Protein restriction specifically decreases the abundance of serum albumin and transthyretin nuclear transcripts in rat liver. J Nutr.

[39] Ingenbleek Y, Young V (1994). Transthyretin (prealbumin) in health and disease: nutritional implications. Annu Rev Nutr.

[40] Moskowitz SR, Pereira G, Spitzer A, Heaf L, Amsel J, Watkins JB (1983). Prealbumin as a biochemical marker of nutritional adequacy in premature infants. J Pediatr.

[41] Thomas MR, Massoudi M, Byrne J, Mitchell MA, Eggert LD, Chan GM (1988). Evaluation of transthyretin as a monitor of protein-energy intake in preterm and sick neonatal infants. J Parenter Enteral Nutr.

[42] Ogunshina SO, Hussain MA (1980). Plasma thyroxine binding prealbumin as an index of mild protein-energy malnutrition in Nigerian children. Am J Clin Nutr.

[43] Devoto G, Gallo F, Marchello C, Racchi O, Garbarini R, Bonassi S, Albalustri G, Haupt E (2006). Prealbumin serum concentrations as a useful tool in the assessment of malnutrition in hospitalized patients. Clin Chem.

[44] Mühlethaler R, Stuck AE, Minder CE, Frey BM (1995). The prognostic significance of protein-energy malnutrition in geriatric patients. Age Ageing.

[45] Waterlow JC (1975). Amount and rate of disappearance of liver fat in malnourished infants in Jamaica. Am J Clin Nutr.

[46] Barbezat GO, Bowie MD, Kaschula RO, Hansen JD (1967). Studies on the small intestinal mucosa of children with protein-calorie malnutrition. S Afr Med J.

[47] Reid M, Badaloo A, Forrester T, Morlese JF, Heird WC, Jahoor F (2002). The acute-phase protein response to infection in edematous and nonedematous protein-energy malnutrition. Am J Clin Nutr.

[48] Meguid MM, Fetissov SO, Varma M, Sato T, Zhang L, Laviano A, Rossi-Fanelli F (2000). Hypothalamic dopamine and serotonin in the regulation of food intake. Nutrition.

[49] McMillan SA, Dickey W, Douglas JP, Hughes DF (2001). Transthyretin values correlate with mucosal recovery in patients with coeliac disease taking a gluten free diet. J Clin Pathol.

[50] Watson F, Dick M (1980). Distribution and inheritance of low serum thyroxine-binding globulin levels in Australian Aborigines: a new genetic variation. Med J Aust.

[51] Bienvenu J, Monneret G, Fabien N, Revillard JP (2000). The clinical usefulness of the measurement of cytokines. Clin Chem Lab Med.

[52] Ingenbleek Y, Bernstein L (1999). The stressful condition as a nutritionally dependent adaptive dichotomy. Nutrition.

[53] Gabay C, Kushner I (1999). Acute-phase proteins and other systemic responses to inflammation. N Engl J Med.

[54] Arnold J, Campbell IT, Samuels TA, Devlin JC, Green CJ, Hipkin LJ, MacDonald IA, Scrimgeour CM, Smith K, Rennie MJ (1993). Increased whole body protein breakdown predominates over increased whole body protein synthesis in multiple organ failure. Clin Sci (Lond).

[55] Murakami T, Ohnishi S, Nishiguchi S, Maeda S, Araki S, Shimada K (1988). Acute-phase response of mRNAs for serum amyloid P component, C-reactive protein and prealbumin (transthyretin) in mouse liver. Biochem Biophys Res Commun.

[56] Banks RE, Forbes MA, Storr M, Higginson J, Thompson D, Raynes J, Illingworth JM, Perren TJ, Selby PJ, Whicher JT (1995). The acute phase protein response in patients receiving subcutaneous IL-6. Clin Exp Immunol.

[57] Myron Johnson A, Merlini G, Sheldon J, Ichihara K, Scientific Division Committee on Plasma Proteins C-PP, International Federation of Clinical ChemistryLaboratory Medicine IFCC (2007). Clinical indications for plasma protein assays: transthyretin (prealbumin) in inflammation and malnutrition. Clin Chem Lab Med.

[58] Cederholm T, Barazzoni R, Austin P, Ballmer P, Biolo G, Bischoff SC, Compher C, Correia I, Higashiguchi T, Holst M, Jensen GL, Malone A, Muscaritoli M, Nyulasi I, Pirlich M, Rothenberg E, Schindler K, Schneider SM, de van der Schueren MA, Sieber C, Valentini L, Yu JC, Van Gossum A, Singer P (2017). ESPEN guidelines on definitions and terminology of clinical nutrition. Clin Nutr.

[59] Evans DC, Corkins MR, Malone A, Miller S, Mogensen KM, Guenter P, Jensen GL, ASPEN Malnutrition Committee (2021). The use of visceral proteins as nutrition markers: An ASPEN position paper. Nutr Clin Pract.

[60] Ingenbleek Y, Richardson SJ, Cody V (2009). Plasma transthyretin reflects the fluctuations of lean body mass. Recent Advances in Transthyretin Evolution, Structure and Biological Functions.

[61] Sergi G, Coin A, Enzi G, Volpato S, Inelmen EM, Buttarello M, Peloso M, Mulone S, Marin S, Bonometto P (2006). Role of visceral proteins in detecting malnutrition in the elderly. Eur J Clin Nutr.

[62] Blackburn GL, Bistrian BR, Maini BS, Schlamm HT, Smith MF (1977). Nutritional and metabolic assessment of the hospitalized patient. J Parenter Enteral Nutr.

[63] Keller U (2019). Nutritional laboratory markers in malnutrition. J Clin Med.

[64] Poulia KA, Yannakoulia M, Karageorgou D, Gamaletsou M, Panagiotakos DB, Sipsas NV, Zampelas A (2012). Evaluation of the efficacy of six nutritional screening tools to predict malnutrition in the elderly. Clin Nutr.

[65] Cederholm T, Jensen GL, Correia MITD, Gonzalez MC, Fukushima R, Higashigushi T, Baptista G, Barazzoni R, Blaauw R, Coats A, Crivelli A, Evans DC, Gramlich L, Fuchs-Tarlovsky V, Keller H, Llido L, Malone A, Mogensen KM, Morley JE, Muscaritoli M, Nyalusi I, Dirlich M, Pisprasert V, de van der Schueren MAE, Siltharm S, Singer P, Tappenden K, Velasco N, Waitzberg D, Yamwong P, Yu J, Van Gossum A, Compher C, GLIM Core Leadership Committee; GLIM Working Group (2019). GLIM criteria for the diagnosis of malnutrition. A consensus report from the global clinical nutrition community. Clin Nutr.

[66] Chiquete E, Ruiz-Sandoval JL, Ochoa-Guzmán A, Sánchez-Orozco LV, Lara-Zaragoza EB, Basaldúa N, Ruiz-Madrigal B, Martínez-López E, Román S, Godínez-Gutiérrez SA, Panduro A (2014). The Quételet index revisited in children and adults. Endocrinol Nutr.

[67] Gavriilidou NN, Pihlsgård M, Elmståhl S (2015). High degree of BMI misclassification of malnutrition among Swedish elderly population: age-adjusted height estimation using knee height and demispan. Eur J Clin Nutr.

[68] Tomiyama AJ, Hunger JM, Nguyen-Cuu J, Wells C (2016). Misclassification of cardiometabolic health when using body mass index categories in NHANES 2005–2012. Int J Obes.

[69] Sedlmeier AM, Baumeister SE, Weber A, Fischer B, Thorand B, Ittermann T, Dörr M, Felix SB, Völzke H, Peters A, Leitzmann MF (2021). Relation of body fat mass and fat-free mass to total mortality: results from 7 prospective cohort studies. Am J Clin Nutr.

[70] Gonzalez MC, Correia MITD, Heymsfield SB (2017). A requiem for BMI in the clinical setting. Curr Opin Clin Nutr Metab Care.

[71] Devakonda A, George L, Raoof S, Esan A, Saleh A, Bernstein LH (2008). Transthyretin as a marker to predict outcome in critically ill patients. Clin Biochem.

[72] Li JD, Xu XF, Han J, Wu H, Xing H, Li C, Yu JJ, Zhou YH, Gu WM, Wang H, Chen TH, Zeng YY, Lau WY, Wu MC, Shen F, Yang T (2019). Preoperative prealbumin level as an independent predictor of long-term prognosis after liver resection for hepatocellular carcinoma: a multi-institutional study. HPB (Oxford).

[73] Han WX, Chen ZM, Wei ZJ, Xu AM (2016). Preoperative pre-albumin predicts prognosis of patients after gastrectomy for adenocarcinoma of esophagogastric junction. World J Surg Oncol.

[74] Ho SY, Guo HR, Chen HH, Peng CJ (2003). Nutritional predictors of survival in terminally ill cancer patients. J Formos Med Assoc.

[75] Isono N, Imamura Y, Ohmura K, Ueda N, Kawabata S, Furuse M, Kuroiwa T (2017). Transthyretin concentrations in acute stroke patients predict convalescent rehabilitation. J Stroke Cerebrovasc Dis.

[76] Dellière S, Pouga L, Neveux N, Hernvann A, De Bandt JP, Cynober L (2021). Assessment of transthyretin cut-off values for a better screening of malnutrition: retrospective determination and prospective validation. Clin Nutr.

[77] Dramaix M, Brasseur D, Donnen P, Bawhere P, Porignon D, Tonglet R, Hennart P (1996). Prognostic indices for mortality of hospitalized children in central Africa. Am J Epidemiol.

[78] Ingenbleek Y (2019). Plasma transthyretin as a biomarker of sarcopenia in elderly subjects. Nutrients.

[79] Liu P, Hao Q, Hai S, Wang H, Cao L, Dong B (2017). Sarcopenia as a predictor of all-cause mortality among community-dwelling older people: a systematic review and meta-analysis. Maturitas.

[80] Chertow GM, Goldstein-Fuchs DJ, Lazarus JM, Kaysen GA (2005). Prealbumin, mortality, and cause-specific hospitalization in hemodialysis patients. Kidney Int.

[81] Bernstein LH, Ingenbleek Y (2002). Transthyretin: its response to malnutrition and stress injury. clinical usefulness and economic implications. Clin Chem Lab Med.

[82] Koike H, Iguchi Y, Sahashi K, Katsuno M (2021). Significance of oligomeric and fibrillar species in amyloidosis: insights into pathophysiology and treatment. Molecules.

[83] Lewis WD, Skinner M, Simms RW, Jones LA, Cohen AS, Jenkins RL (1994). Orthotopic liver transplantation for familial amyloidotic polyneuropathy. Clin Transplant.

[84] Suhr OB, Conceição IM, Karayal ON, Mandel FS, Huertas PE, Ericzon BG (2014). Post hoc analysis of nutritional status in patients with transthyretin familial amyloid polyneuropathy: impact of tafamidis. Neurol Ther.

[85] Sekijima Y, Dendle MA, Kelly JW (2006). Orally administered diflunisal stabilizes transthyretin against dissociation required for amyloidogenesis. Amyloid.

[86] Coelho T, Adams D, Silva A, Lozeron P, Hawkins PN, Mant T, Perez J, Chiesa J, Warrington S, Tranter E, Munisamy M, Falzone R, Harrop J, Cehelsky J, Bettencourt BR, Geissler M, Butler JS, Sehgal A, Meyers RE, Chen Q, Borland T, Hutabarat RM, Clausen VA, Alvarez R, Fitzgerald K, Gamba-Vitalo C, Nochur SV, Vaishnaw AK, Sah DWY, Gollob JA, Suhr OB (2013). Safety and efficacy of RNAi therapy for transthyretin amyloidosis. N Engl J Med.

[87] Benson MD, Waddington-Cruz M, Berk JL, Polydefkis M, Dyck PJ, Wang AK, Planté-Bordeneuve V, Barroso FA, Merlini G, Obici L, Scheinberg M, Brannagan TH, Litchy WJ, Whelan C, Drachman BM, Adams D, Heitner SB, Conceição I, Schmidt HH, Vita G, Campistol JM, Gamez J, Gorevic PD, Gane E, Shah AM, Solomon SD, Monia BP, Hughes SG, Kwoh TJ, McEvoy BW, Jung SW, Baker BF, Ackermann EJ, Gertz MA, Coelho T (2018). Inotersen treatment for patients with hereditary transthyretin amyloidosis. N Engl J Med.

[88] Magrinelli F, Fabrizi GM, Santoro L, Zanette G, Cavallaro T, Tamburin S (2020). Pharmacological treatment for familial amyloid polyneuropathy. Cochrane Database. Syst Rev.

[89] Tomson R, Fridolin I, Luman M (2015). Lean body mass assessment based on UV absorbance in spent dialysate and dual-energy X-ray absorptiometry. Int J Artif Organs.

[90] Player EL, Morris P, Thomas T, Chan WY, Vyas R, Dutton J, Tang J, Alexandre L, Forbes A (2019). Bioelectrical impedance analysis (BIA)-derived phase angle (PA) is a practical aid to nutritional assessment in hospital in-patients. Clin Nutr.

[91] Cui N, Tong H, Li Y, Ge Y, Shi Y, Lv P, Zhao X, Zhang J, Fu G, Zhou Y, Jiang K, Lin N, Bai T, Jin R, Wei S, Yang X, Li X (2021). Role of prealbumin in predicting the prognosis of severely and critically ill Covid-19 patients. Am J Trop Med Hyg.

[92] Luo Y, Xue Y, Mao L, Yuan X, Lin Q, Tang G, Song H, Wang F, Sun Z (2020). Prealbumin as a predictor of prognosis in patients with coronavirus disease 2019. Front Med (Lausanne).

[93] Chen R, Li L, Li C, Su Y, Zhang Y, Pang X, Zheng J, Zeng Z, Chen MH, Zhang S (2021). Prealbumin and retinol-binding protein 4: The promising inflammatory biomarkers for identifying endoscopic remission in Crohn’s disease. J Inflamm Res.

[94] Fan Y, Sun Y, Man C, Lang Y (2021). Perioperative serum prealbumin level and adverse prognosis in patients with hepatocellular carcinoma after hepatectomy: a meta-analysis. Front Oncol.

[95] Miura T, Amano K, Shirado A, Baba M, Ozawa T, Nakajima N, Suga A, Matsumoto Y, Shimizu M, Shimoyama S, Kuriyama T, Matsuda Y, Iwashita T, Mori I, Kinoshita H (2018). Low transthyretin levels predict poor prognosis in cancer patients in palliative care settings. Nutr Cancer.

[96] Shimura T, Shibata M, Inoue T, Owada-Ozaki Y, Yamaura T, Muto S, Hasegawa T, Shio Y, Suzuki H (2019). Prognostic impact of serum transthyretin in patients with non-small cell lung cancer. Mol Clin Oncol.

[97] Akashi M, Minami Y, Haruki S, Jujo K, Hagiwara N (2019). Prognostic implications of prealbumin level on admission in patients with acute heart failure referred to a cardiac intensive care unit. J Cardiol.

[98] Sato S, Shiozawa M, Nukada S, Iguchi K, Kazama K, Atsumi Y, Numata M, Tamagawa H, Tanaka K, Oshima T, Rino Y (2021). Preoperatve pre-albumin concentration as a predictor of short-term outcomes in elderly patients with colorectal cancer. Anticancer Res.

[99] Kumagai E, Hosohata K, Furumachi K, Takai S. Range of serum transthyretin levels in hemodialysis patients at a high risk of 1-year mortality: a retrospective cohort study. Ther Apher Dial 2021; [Epub ahead of print] doi:10.1111/1744-9987.13768 10.1111/1744-9987.13768 34845841

[100] Yang HT, Yim H, Cho YS, Kim D, Hur J, Kim JH, Lee BC, Seo DK, Kim HS, Chun W (2012). Prediction of clinical outcomes for massively-burned patients via serum transthyretin levels in the early postburn period. J Trauma Acute Care Surg.

[101] Lee KH, Cho JH, Kwon O, Kim SU, Kim RH, Cho YW, Jung HY, Choi JY, Kim CD, Kim YL, Park SH (2016). Low prealbumin levels are independently associated with higher mortality in patients on peritoneal dialysis. Kidney Res Clin Pract.

[102] Bae HJ, Lee HJ, Han DS, Suh YS, Lee YH, Lee HS, Cho JJ, Kong SH, Yang HK (2011). Prealbumin levels as a useful marker for predicting infectious complications after gastric surgery. J Gastrointest Surg.

[103] Akbar MR, Pranata R, Wibowo A, Lim MA, Sihite TA, Martha JW (2021). The association between serum prealbumin and poor outcome in COVID-19—systematic review and meta-analysis. Eur Rev Med Pharmacol Sci.

[104] Seesen M, Sirikul W, Ruangsuriya J, Griffiths J, Siviroj P (2021). Cognitive frailty in Thai community-dwelling elderly: prevalence and its association with malnutrition. Nutrients.

[105] Sugumar D, Arockiaraj J, Amritanand R, David KS, Krishnan V (2021). Role of biochemical nutritional parameters as predictors of postoperative morbidity in major spine surgeries. Asian Spine J.

[106] Shahriari M, Rezaei E, Bakht LA, Abbasi S (2015). Comparison of the effects of enteral feeding through the bolus and continuous methods on blood sugar and prealbumin levels in ICU inpatients. J Educ Health Promot.

[107] Zinellu A, Mangoni AA (2021). Serum prealbumin concentrations, Covid-19 severity, and mortality: a systematic review and meta-analysis. Front Med (Lausanne).

